# Molecular defense strategy of volatile organic compound-emitting plants (order Piperales) against herbivorous mammals

**DOI:** 10.1038/s42003-025-09273-4

**Published:** 2025-12-01

**Authors:** Huajun Cai, Deyuan Su, Anna Luo, Yalan Han, Hao Zhang, Peter Muiruri Kamau, Haiying Wu, Ren Lai, Lei Luo

**Affiliations:** 1 School of Life Sciences, Division of Life Sciences and Medicine, University of Science and Technology of China, Hefei, China; 2 Engineering Laboratory of Peptides of Chinese Academy of Sciences, Key Laboratory of Bioactive Peptides of Yunnan Province, KIZ-CUHK Joint Laboratory of Bioresources and Molecular Research in Common Diseases, National Resource Center for Non-Human Primates, National Research Facility for Phenotypic & Genetic Analysis of Model Animals (Primate Facility), State Key Laboratory of Genetic Evolution & Animal Models, Sino-African Joint Research Center, and New Cornerstone Science Laboratory, Kunming Institute of Zoology, The Chinese Academy of Sciences, Kunming, China; 3 University of Chinese Academy of Sciences, Beijing, China; 4 Department of Emergency, First Affiliated Hospital of Kunming Medical University, Kunming, China; 5 Center for Evolution and Conservation Biology, Southern Marine Science and Engineering Guangdong Laboratory (Guangzhou), Guangzhou, China

**Keywords:** Ligand-gated ion channels, Ion channels in the nervous system

## Abstract

Plants have evolved diverse strategies to defend against herbivores, including structural barriers such as trichomes and tough leaves, the production of toxic secondary metabolites, the emission of volatile organic compounds (VOCs), and the recruitment of natural predators to deter herbivory. However, the molecular mechanisms underlying their ability to deter large herbivorous mammals remain poorly understood. In this study, we demonstrate that the order Piperales, which is particularly rich in VOCs, employs a conserved chemical defense strategy targeting herbivorous mammals. Behavioral assays, transgenic models, and electrophysiological analyses revealed that VOCs from Piperales species, particularly *Houttuynia cordata*, activate TRPA1—a sensory ion channel critical for detecting irritants—in mice and herbivores such as cattle and goats. A stable derivative of the key VOC houttuynin, sodium houttuyfonate (SH), selectively activated TRPA1 by binding conserved cysteine residues, triggering aversion in herbivorous mammals. Crucially, TRPA1 activation sites upon VOC application were conserved across herbivorous species, suggesting that Piperales plants employ a conserved evolutionary strategy to defend against herbivorous mammals. Our findings reveal a compelling case of lineage-specific defensive adaptation within Piperales, providing novel insights into plant-herbivore interactions. This research deepens our understanding of the critical role of chemical defenses in plant survival, adaptation, and ecological niche specialization.

## Abstract (web summary)

Volatile organic compounds from plants in the order Piperales (such as *Houttuynia cordata*) activate the TRPA1 ion channels of herbivorous mammals like cattle and goats and trigger aversion reactions in these animals, suggesting a conserved defense strategy among Piperales plants.

## Introduction

The defense mechanisms employed by plants to counter herbivory are pivotal for their survival, reproduction, and ecological persistence. The ongoing evolutionary arms race between plants and herbivores has profoundly influenced the adaptive strategies of both groups, driving complex co-evolutionary dynamics^1,2^. As primary producers, plants serve as essential sources of energy and nutrients for herbivores^3^, resulting in the concurrent evolution of diverse defense strategies to deter herbivory. The defense strategies employed by plants against herbivores represent a complex and highly adaptive process, integrating physical, chemical, and biological mechanisms^4,5^. Physical defenses, including structures such as thorns, spines, and trichomes, act as barriers that inhibit herbivores from feeding^6^. Chemical defenses include the synthesis of secondary metabolites such as alkaloids, cyanogenic glycosides, glucosinolates, and terpenoids, which exert toxic, repellent, or anti-nutritional effects on herbivores^5^. Beyond these direct defenses, plants also employ volatile organic compounds (VOCs), which play dual roles in plant-herbivore interactions. VOCs can act as deterrents by repelling herbivores or inducing toxic effects, while simultaneously functioning as chemical signals that attract the natural enemies of herbivores, such as parasitic wasps and predatory insects^7–9^.

Advancements in plant defense research have revealed that plants dynamically adjust their defensive responses to herbivore attack patterns^10^. Evidence suggests that plants can modify their defense strategies to align with typical herbivore feeding behaviors, demonstrating both specificity and adaptability^10^. Despite these insights, critical gaps remain in our understanding of plant defense mechanisms. Notably, research has disproportionately focused on small herbivores, with studies examining the interactions between plants and large herbivores remaining relatively limited^11^. Additionally, many existing studies have emphasized pairwise interactions between plants and herbivores, resulting in a critical gap in understanding the broader patterns and variations in plant defense strategies across species and ecosystems. Establishing a comprehensive framework to address these gaps is essential for advancing knowledge of the complexity and diversity of plant defense mechanisms against herbivores.

This study examined whether the release of VOCs represents a conserved defensive strategy among plants to deter herbivorous mammals. Species within the order Piperales, recognized for their abundance and diversity of VOCs, were investigated for their potential deterrent effects on herbivores. Results demonstrated that TRPA1 activation likely functions as a universal mechanism utilized by plants such as *Houttuynia cordata* (*H. cordata*) within the order Piperales to defend against herbivorous mammals. These findings demonstrate a conserved evolutionary mechanism in the chemical defenses of Piperales, offering valuable insights into the complex interaction mechanisms between plants and herbivorous mammals.

## Results

### Piperales exhibits high richness in VOC-emitting plants

**Fig. 1:**
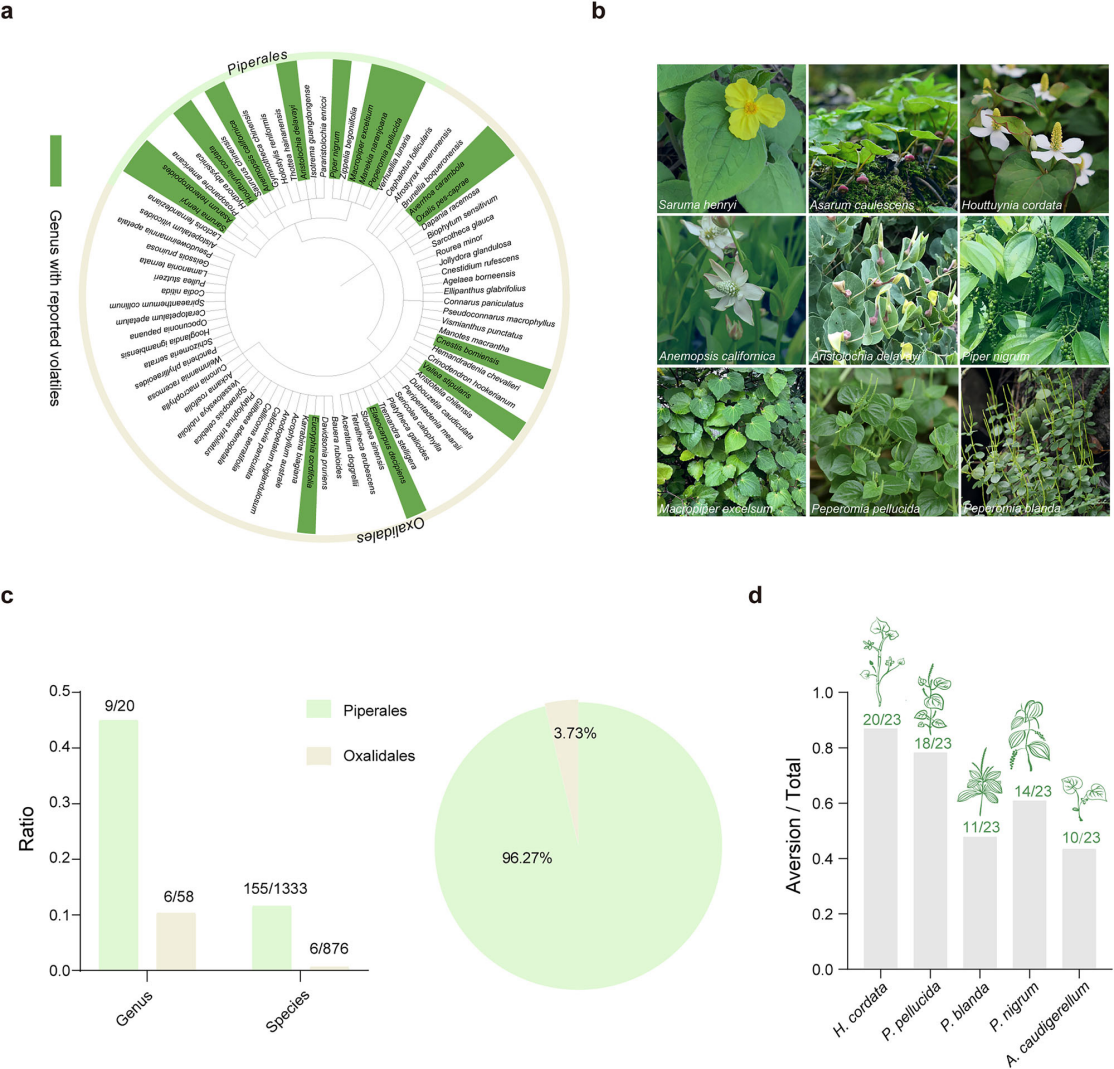
Statistical overview of volatile plants in the order Piperales. **a** Phylogenetic tree of representative plants from the orders Piperales and Oxalidales. Dark green represents volatile plants; light green represents Piperales plants; light yellow represents Oxalidales plants. **b** Representative volatile plants in the order Piperales. **c** Ratio of volatile plants to total plants at the species and genus levels (Column Chart). Relative proportion of volatile plants in the orders Piperales and Oxalidales (Pie Chart). **d** Comparison of feeding trials (in parenthesis) with leaves from representative volatile plants. Twenty-three goats were tested with all leaf types twice to ensure reproducibility. Values preceding the dividing line represent the number of plant rejections by goats, while values following it denote the total presentations administered.

To investigate VOC-mediated chemical defenses, this study focused on Piperales, renowned for its exceptional abundance and diversity of VOC-emitting plants^12,13^. This order encompasses three families and a wide variety of species, including *Saruma henryi*, *Anemopsis californica*, *Piper nigrum*, and *H. cordata* (Fig. 1a, b). These species are well-known representatives of plants that actively emit VOCs. A systematic survey of 1333 species within Piperales revealed that ~11.63% (155 species) are capable of producing VOCs. In contrast, a comparative analysis of 876 species of the order Oxalidales showed that only 0.68% (6 species) emit VOCs. Genus-level analysis identified similar disparity, with 45% of genera in Piperales (9 out of 20 genera) containing VOC-emitting species compared to 10.34% of genera in Oxalidales (6 out of 58 genera) (Fig. 1c). Statistical analysis of VOC-emitting plants across these two orders demonstrated a stark contrast, with 96.27% of the surveyed VOC-emitting plants belonging to Piperales, while only 3.73% were from Oxalidales (Fig. 1c). This pattern persisted in our expanded comparison with Saxifragales, where Piperales exhibited an 8-fold greater production of herbivore-deterrent VOCs (Supplementary Fig. 1). Behavioral assays further supported these findings, with herbivorous goats exhibiting typical aversion to representative VOC-emitting plants in Piperales (Fig. 1d). These results highlight the remarkable enrichment of VOC-emitting plants within the order Piperales, suggesting a key evolutionary adaptation for herbivore deterrence through chemical defense mechanisms.

### VOCs activate a subset of somatosensory neurons

**Fig. 2:**
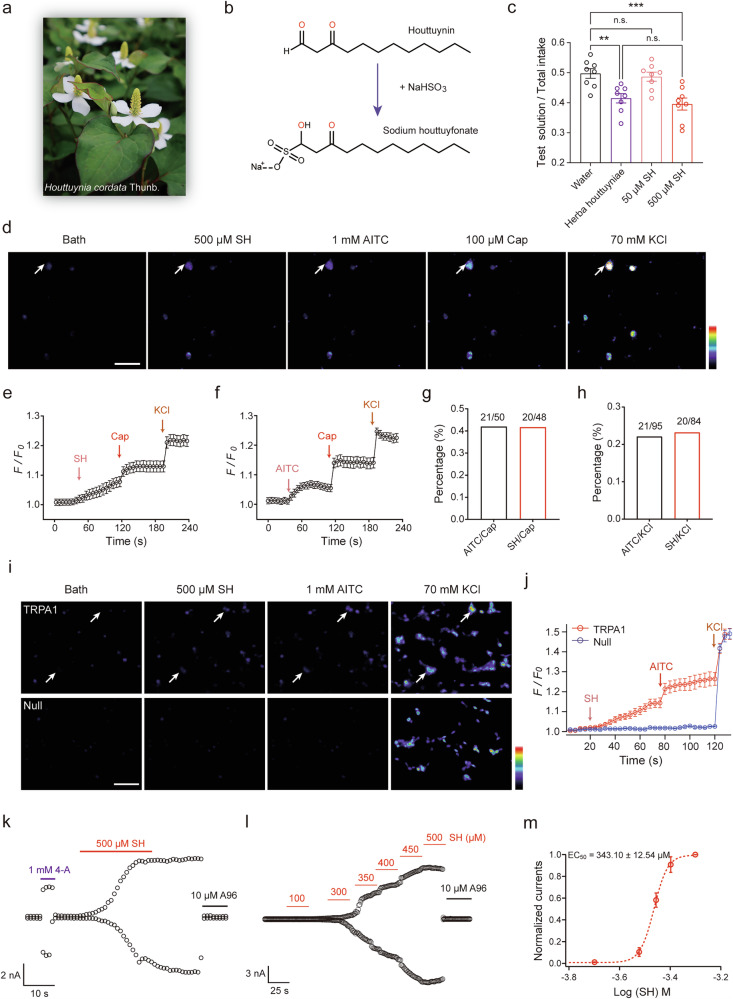
SH selectively targets the TRPA1 channel. **a** *H. cordata* is a perennial herbaceous plant widely recognized across Japan, Korea, China, and North-East India. **b** Chemical structures of houttuynin and SH. **c** Relative preference in WT mice for water compared with water to which crude *H. cordata* extract (1 mg/mL, *n* = 8) and SH (50 and 500 μM, *n* = 8 for each group) at the concentrations indicated in two-bottle taste preference tests. Data represent individual animals and mean ± SEM. Statistical analysis was performed using one-way ANOVA (*F* = 9.563) and Bonferroni’s *post hoc* test: water versus crude *H. cordata* extract (*t* = 3.699, *p* = 0.003); water versus 50 μM SH (*t* = 0.5626*, p* > 0.9999); water versus 500 μM SH (*t* = 4.342, *p* = 0.0007); Herba houttuyniae versus 500 μM SH (*t* = 0.6430, *p* > 0.9999). **p < 0.01, ***p < 0.001, n.s. no significant difference. **d** Calcium imaging of cultured trigeminal ganglion (TG) neurons exposed sequentially to 500 μM SH, 1 mM allyl isothiocyanate (AITC), 100 μM capsaicin (Cap), and 70 mM potassium chloride (KCl). SH responses were observed in AITC-positive neurons (Arrow). Scale bar, 100 μm. **e** Averaged intracellular Ca^2+^ increases in cultured mouse TG neurons following consecutive applications of 500 μM SH, 10 μM Cap, and 70 mM KCl (*n* = 19). **f** Average intracellular Ca^2+^ increases in cultured mouse TG neurons in response to consecutive applications of 100 μM AITC, 10 μM Cap, and 70 mM KCl (*n* = 11). **g** Percentage of AITC- or SH-sensitive neurons in Cap-positive neurons, with ratios displayed above bars. **h** Percentage of AITC- or SH-sensitive neurons in KCl-positive neurons, with ratios displayed above bars. **i** Calcium imaging of HEK293T cells expressing TRPA1 or null controls exposed sequentially to SH (500 μM), AITC (1 mM), and KCl (70 mM). Scale bar, 100 μm. **j** Representative calcium fluorescence signals of HEK293T cells expressing TRPA1 or null controls, based on data from 5 cells. **k** Whole-cell TRPA1 currents elicited by the application of 1 mM 4-A and 500 μM SH. **l** Whole-cell TRPA1 currents elicited by the application of different doses of SH (100, 300, 350, 400, 450, and 500 μM). **m** Dose-response relationships for SH fitted to the Hill equation. Half-maximal response concentration (EC_50_) and Hill coefficient (mean ± SEM) were 343.10 ± 12.54 μM and 1.93, respectively (*n* = 5).

To investigate the molecular mechanisms underlying VOC-induced chemical defenses in the order Piperales, this study focused on *H. cordata*, a plant noted for its pungent and irritating taste, which demonstrated the strongest repellent effects against herbivores among the tested species (Figs. 1d and 2a). With its high VOC content, established role in plant defense, and availability of genomic resources^14,15^, *H. cordata* was selected as an ideal model for studying VOC-mediated herbivore deterrence. Among its essential oils, houttuynin is considered to be the main compound associated with its characteristic irritating taste^16^. However, due to the instability of houttuynin and its tendency to polymerize during extraction, sodium houttuyfonate (SH)^17^, a stable derivative retaining the same pungency as houttuynin (Fig. 2b), was used for subsequent experiments.

To explore the role of houttuynin in chemical defense, two-bottle taste preference test were conducted to evaluate the aversive effects of crude *H. cordata* extract and SH in wild-type (WT) mice (Fig. 2c). The results revealed that mice exposed to SH consumed significantly less tested solution compared to water, with Bonferroni’s *post hoc* analysis confirming reduced consumption in the 500 μM SH group (0.396 ± 0.020, *n* = 8) and crude *H. cordata* extract group (0.415 ± 0.016, *n* = 8) compared to the water group (0.497 ± 0.016, *n* = 8). Furthermore, the repellent activity of the crude *H. cordata* extract (1 mg/mL) was comparable to that of 500 μM SH, indicating that SH effectively mimics the aversive properties of houttuynin and serves as a robust candidate for studying VOC-induced aversion.

The trigeminal somatosensory system, which plays a critical role in the chemosensation and perception of VOCs^18^, was analyzed to determine whether SH activates sensory neurons in the mouse trigeminal ganglia (TG). Calcium imaging revealed that SH (500 μM) induced Ca^2+^ influx in a distinct subset of TG neurons in adult mice (Fig. 2d). Notably, most SH-sensitive neurons also responded to allyl isothiocyanate (AITC) and capsaicin (Cap), specific agonists for the TRPA1 and TRPV1 channels^19,20^, respectively. Using 70 mM KCl as a positive control, intracellular calcium increases were observed in all excitable neurons (Fig. 2d–f). Given that TRPA1 and TRPV1 are highly co-expressed in the TG^21–23^, our findings demonstrated that all SH-sensitive neurons responded similarly to Cap and KCl, as all AITC-sensitive neurons did (Fig. 2e, f). Furthermore, ~42.0% of SH-sensitive neurons responded to Cap, and 22.1% responded to 70 mM KCl, closely aligning with the percentages observed for AITC-sensitive neurons (41.7% and 21.3%, respectively) (Fig. 2g, h). These findings demonstrate that SH activates a specific subset of TRPA1 and TRPV1-expressing sensory neurons, highlighting its potential role in triggering somatosensory responses integral to VOC-mediated herbivore deterrence.

### SH selectively activates TRPA1 channels

To identify the molecular targets involved in SH-induced sensory responses, the interaction of SH with somatic sensory neurons was investigated. Previous studies have demonstrated that TRPA1 and TRPV1 are critical sensory targets in TG neurons^21^. The consistent activation of AITC- and Cap-sensitive neurons by SH suggested that the TRPA1 and TRPV1 channels were likely candidates for SH-mediated effects. TRPA1 is notably expressed in both the geniculate ganglion and TG, which consist primarily of sensory neurons responsible for detecting environmental stimuli^19,24^. Therefore, to assess whether TRPA1 functions as an endogenous target in SH sensing, calcium imaging was performed on HEK293T cells heterologously expressing TRPA1. Results indicated that SH (500 μM) elicited a typical calcium influx in AITC-responding cells but not in mock-transfected cells (Fig. 2i, j). These results indicated that SH activates TRPA1 channels in both endogenous and heterologous systems. TRPA1 Whole-cell patch-clamp recordings confirmed the direct activation of TRPA1 by SH in HEK293T cells, with perfusion of 500 μM SH eliciting a larger current than 4-aminodiphenylamine (4-A), a reversible TRPA1 agonist^25^. This response was blocked by 10 μM A-967079 (A-96), a selective TRPA1 inhibitor^25^ (Fig. 2k). SH further activated TRPA1 in a dose-dependent manner, with an EC_50_ value of 343.10 ± 12.54 μM (*n* = 6) and a Hill coefficient of 1.93 (Fig. 2l, m). The specificity of SH for TRPA1 was assessed by examining its effects on other ion channels implicated in chemosensation^26^, including TRPV1, TRPV2, TRPV3, and TRPM8. Results indicated that SH (500 μM) had no apparent effect on HEK293T cells expressing TRPV1, TRPV2, TRPV3, or TRPM8, but Cap (10 μM), 2-APB (1 mM or 100 μM), or menthol (1 mM) which are TRPV1, TRPV2, TRPV3 or TRPM8 agonists respectively, induced specific whole-cell currents in cells expressing those channels respectively (Supplementary Fig. 2a–e). Similarly, SH exhibited no activity on other chemosensory channels^27,28^, such as ASICs or OTOP1 (Supplementary Fig. 2g, h). Collectively, these findings confirm the high selectivity of SH for TRPA1.

Single-channel recordings in the outside-out configuration were conducted to explore the mechanism of SH-mediated TRPA1 activation. Application of 500 μM SH significantly increased the frequency and duration of channel opening events, consistent with a potentiation effect on gating (Supplementary Fig. 3a, b). However, 500 μM SH had minimal impact on the amplitude of single-channel currents. The single-channel outward current of TRPA1 was 12.54 ± 0.29 pA (*n* = 5) in SH-free solution, and 12.60 ± 0.37 pA (*n* = 5) in the presence of 500 μM SH (*n* = 5; Supplementary Fig. 3c, d). These results suggest that SH potentiates TRPA1 activity by increasing the frequency of channel opening without affecting conductance. Overall, the findings establish SH as a highly selective agonist of TRPA1, directly modulating its gating properties to mediate sensory responses. This specificity highlights the critical role of TRPA1 in SH-induced chemosensory signaling.

### SH targets TRPA1 to induce aversive behavior in mice

**Fig. 3:**
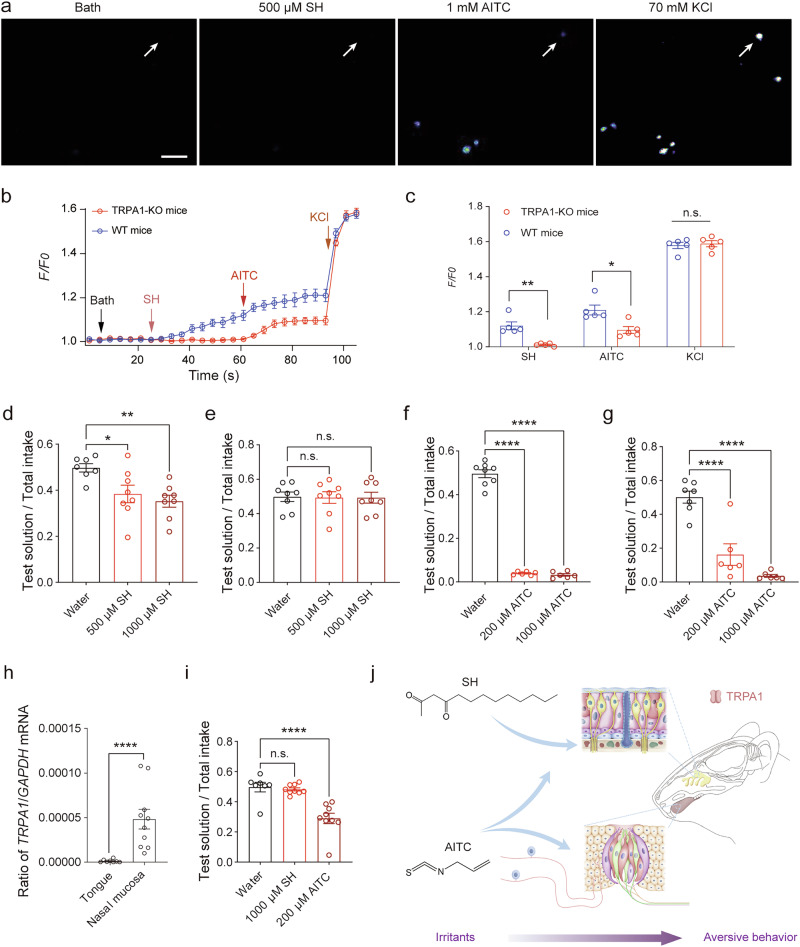
SH induces aversion behavior in mice by targeting nasal TRPA1 channels. **a** Calcium imaging of TG neurons from TRPA1-KO mice challenged sequentially with SH (500 μM), AITC (1 mM), and KCl (70 mM). Scale bar, 100 μm. **b**, **c** Representative calcium fluorescence signals of TG neurons from wild-type (WT) and TRPA1-KO mice (*n* = 5 per trace). *p < 0.05, **p < 0.01,  n.s. no significant difference. **d**, **e** Relative water preference in WT and TRPA1-KO mice during two-bottle taste preference tests with SH at indicated concentrations (*n* = 7 ~ 8). Statistical analysis for WT: one-way ANOVA, *F* = 6.413; water vs. 500 μM SH: *t* = 2.687, *p* = 0.0283; water vs. 1000 μM SH: *t* = 3.436, *p* = 0.0052. For TRPA1-KO: *F* = 0.0091; water vs. 500 μM SH: *t* = 0.1090, *p* > 0.9999; water vs. 1 000 μM SH: *t* = 0.1228, *p* > 0.9999. *p < 0.05, **p < 0.01, n.s. no significant difference. Data are presented as mean ± SEM. **f**, **g** Relative water preference in WT and TRPA1-KO mice during two-bottle taste preference test with AITC at indicated concentrations (*n* = 6 ~ 8). Statistical analysis for WT: one-way ANOVA, *F* = 434.6; water vs. 200 μM AITC: *t* = 24.72, *p* < 0.0001; water vs. 1000 μM AITC: *t* = 25.11, *p* < 0.0001. For TRPA1-KO: *F* = 34.69; water vs. 200 μM AITC: *t* = 5.815, *p* < 0.0001; water vs. 1 000 μM AITC: *t* = 7.974, *p* < 0.0001. ****p < 0.0001. Data are presented as mean ± SEM. **h** TRPA1 gene expression in nasal mucosa (*n* = 10) and tongue (*n* = 8) of mice. **i** Relative water preference in olfactory-deprived mice exposed to 1000 μM SH (*n* = 9) and 200 μM AITC (*n* = 9) in two-bottle taste preference test. **j** Model depicting SH detection mediated by nasal TRPA1 channels. n.s. no significant difference.

To investigate whether SH directly activates the TRPA1 channel in vivo, experiments were conducted using *Trpa1* knock-out (TRPA1-KO) mice. TRPA1-KO mice, widely employed to study the physiological role of TRPA1 in various sensory and pathophysiological processes, including odor perception^29,30^, were genetically verified by polymerase chain reaction (PCR) (Supplementary Fig. 4). Single-cell calcium imaging of TG neurons from TRPA1-KO mice revealed that, unlike WT mice, the application of 500 μM SH did not induce a significant increase in intracellular Ca^2+^ levels (Fig. 3a–c). However, exposure to 70 mM KCl elicited robust intracellular calcium signals in both WT and TRPA1-KO neurons (Fig. 3a–c). These results indicate that SH specifically targets the TRPA1 channel, as its effects were absent in TRPA1-KO TG neurons.

Behavioral assays further confirmed the physiological role of TRPA1 in mediating SH-induced aversive responses. In WT mice, a two-bottle taste preference test demonstrated significant avoidance behaviors in response to SH. Notably, both 500 and 1000 μM SH significantly reduced water consumption compared to vehicle controls (Fig. 3d). Specifically, the 1000 μM SH group consumed a smaller proportion of water (0.35 ± 0.03, *n* = 8) than the 500 μM SH group (0.38 ± 0.04, *n* = 8) and the control group (0.50 ± 0.02, *n* = 7). Notably, the avoidance behaviors observed in WT mice were absent in TRPA1-KO mice when exposed to either 500 μM SH or 1000 μM SH (Fig. 3e). These findings demonstrate that SH exerts its aversive effects in mice by selectively targeting the TRPA1 channel, which is highly expressed in chemosensory neurons. The absence of SH-induced calcium signaling and avoidance behavior in TRPA1-KO mice underscores the critical role of TRPA1 in mediating the sensory and behavioral responses elicited by SH.

### SH activates TRPA1 in the nasal mucosa

The aversive effects of SH were compared to those of other TRPA1 agonists, including AITC, in a comparable two-bottle taste preference test. Results indicated that AITC induced significant avoidance behaviors in WT mice at both tested concentrations (200 and 1000 μM), comparable to the responses elicited by SH (Fig. 3f). However, in TRPA1-KO mice, significant aversion to AITC persisted at both concentrations, albeit with a reduced response at 200 μM (Fig. 3g). These findings suggest that AITC may interact with additional targets beyond TRPA1, whereas SH exhibits a more specific aversive effect through TRPA1 activation.

The trigeminal nerve, which innervates the nasal mucosa, oral cavity, and ocular tissues, plays a central role in mediating chemosensory responses in mammals^31^. Further analysis revealed that TRPA1 was predominantly expressed in the nasal mucosa, with minimal expression in the oral cavity (Fig. 3h). This distribution aligns with the observation that SH activity was nearly abolished in TRPA1-KO mice. The specificity of SH for nasal TRPA1 was further supported by experiments using an olfactory deprivation model in mice. In this model, aversion responses to high concentrations of SH were almost entirely eliminated, while responses to AITC were only mildly attenuated (Fig. 3i and Supplementary Fig. 5). These findings confirm that SH is perceived primarily through TRPA1 channels in the nasal mucosa, highlighting the role of TRPA1 as a key mediator of SH-induced aversion (Fig. 3j).

### SH targets the intracellular allosteric nexus of TRPA1

**Fig. 4:**
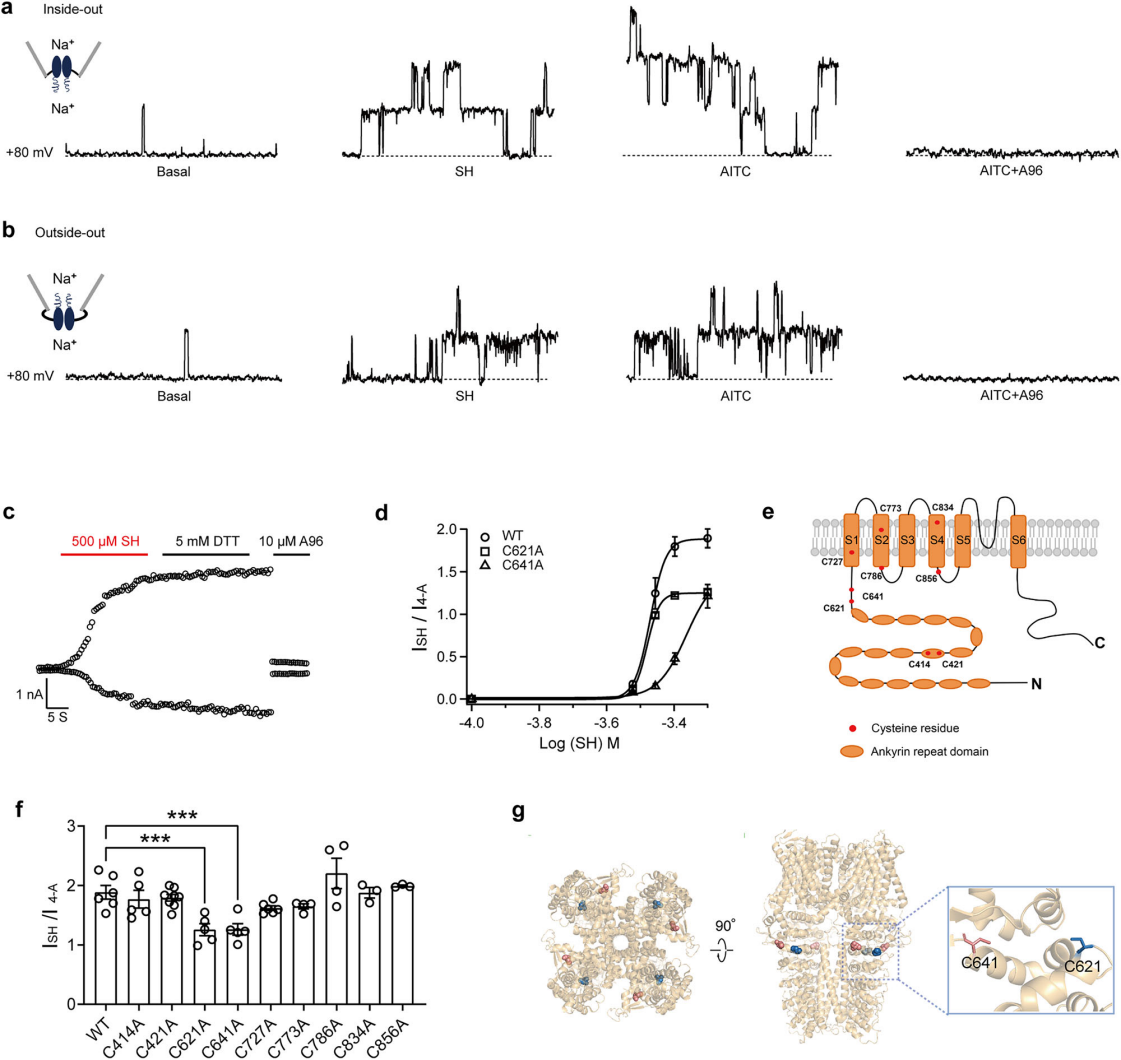
SH binds the intracellular allosteric nexus of TRPA1. Patch-clamp recordings of single TRPA1 channels excised HEK cell patches, showing responses in outside-out (**a**) and inside-out (**b**) configurations to bath application of SH (500 μM), AITC (1 mM), and A96 (10 μM, A967079). Identity of TRPA1 channels was confirmed by application of selective TRPA1 antagonist, A967079. **c** Whole-cell TRPA1 currents elicited by 500 μM SH, 5 mM dithiothreitol, and 10 μM A96. **d** Dose-response relationships of WT and mutant TRPA1 channels for SH, fitted to the Hill equation and compared to 4-A induced currents (*n* = 5). **e**, **g** Structural model of TRPA1 structure and close-up view of SH binding site. **f** Normalized currents calculated as the ratio of SH-induced currents to 4-A-induced currents in response to 500 μM SH in HEK293 cells expressing WT hTRPA1 or hTRPA1 mutants (*n* = 3 ~ 8). Statistical analysis was conducted using one-way ANOVA (*F* = 6.889), followed by Sidak’s multiple comparisons test: WT vs. C621A, *t* = 4.271, *p* = 0.0002; WT vs. C641A, *t* = 4.250, *p* = 0.0003. *p < 0.05, **p < 0.01, ***p < 0.001. Data are presented as mean ± SEM.

Given the established role of SH as a volatile compound mediating plant defense, we next investigated how SH directly activates TRPA1. Whole-cell patch-clamp recordings revealed that extracellular application of SH induced TRPA1 currents in a gradual and sustained manner (Fig. 2j, l), suggesting that extracellular SH crosses the plasma membrane to activate TRPA1 from the intracellular side. To confirm this hypothesis, SH was applied to excised membranes in both outside-out and inside-out configurations. Remarkably, TRPA1 channel activation was consistently observed in both configurations, mirroring the effects of AITC, a membrane-permeable TRPA1 agonist (Fig. 4a, b). These findings indicate that SH exerts its effects through intracellular interaction with TRPA1 channels, requiring membrane permeability to reach its site of action.

Interestingly, SH-induced TRPA1 current exhibited irreversible characteristics, as they persisted even after perfusion with SH-free bath solutions. This irreversible activation is reminiscent of covalent modification mechanisms seen in TRPA1 activation by other electrophilic compounds. For example, cinnamaldehyde activates TRPA1 via Michael addition to cysteines^32^, while 2-aminoethylmethanethiosulphonate (MTSEA) and iodoacetamide modify cysteines via disulfide bonds and alkylation, respectively^33,34^. Given the similarity between SH-induced irreversible activation of TRPA1 and the activation mechanisms of other electrophiles, we hypothesized that SH may activate TRPA1 through covalent binding to cysteines. The structural resemblance between SH and MTSEA, a compound known to form disulfide bonds with cysteines, further supported this possibility. To evaluate the hypothesis, we employed the cell-permeable reducing agent dithiothreitol (DTT). DTT is known for its specificity in reversing disulfide modifications of cysteine residues without interfering with other forms of covalent interactions, including those arising from Michael addition or cysteine conjugation mechanisms^34,35^. Following the application of 5 mM DTT to SH-induced whole-cell TRPA1 currents^34^, no apparent reduction in current amplitude was observed (Fig. 4c), indicating that SH covalently modification of cysteines of TRPA1 in other fashion distinct from those mediated by MTSEA.

To identify specific cysteine residues involved in SH binding, mutations were introduced to key amino acid residues crucial for irreversible covalent modification^36^. Mutation of C641 significantly reduced SH binding affinity, yielding an EC_50_ value of 431.68 ± 9.72 μM (*n* = 6), while mutation of C621 reduced the maximal open probability without affecting binding affinity (Fig. 4d). Systematic testing of other cysteine residues located in the transmembrane and intracellular regions revealed no significant difference in SH sensitivity compared to the WT channel, except for C621 and C641 (Fig. 4f). We further generated the C621A/C641A double mutant, which significantly reduced SH affinity (Supplementary Fig. 6). Although SH affinity for C621A/C641A showed no significant difference versus single mutants, its functional response amplitude was lower than either C621A or C641A alone. Given the double mutant’s inability to fully abolish SH effects, these results suggest that—in addition to direct interactions with C621/C641—other sites contribute to SH binding on TRPA1. Collectively, these findings indicate that SH binds directly to TRPA1, likely interacting with potential binding pockets in which SH could nestle (Fig. 4e, g).

### *Houttuynia cordata* leaf scent deters herbivores

**Fig. 5:**
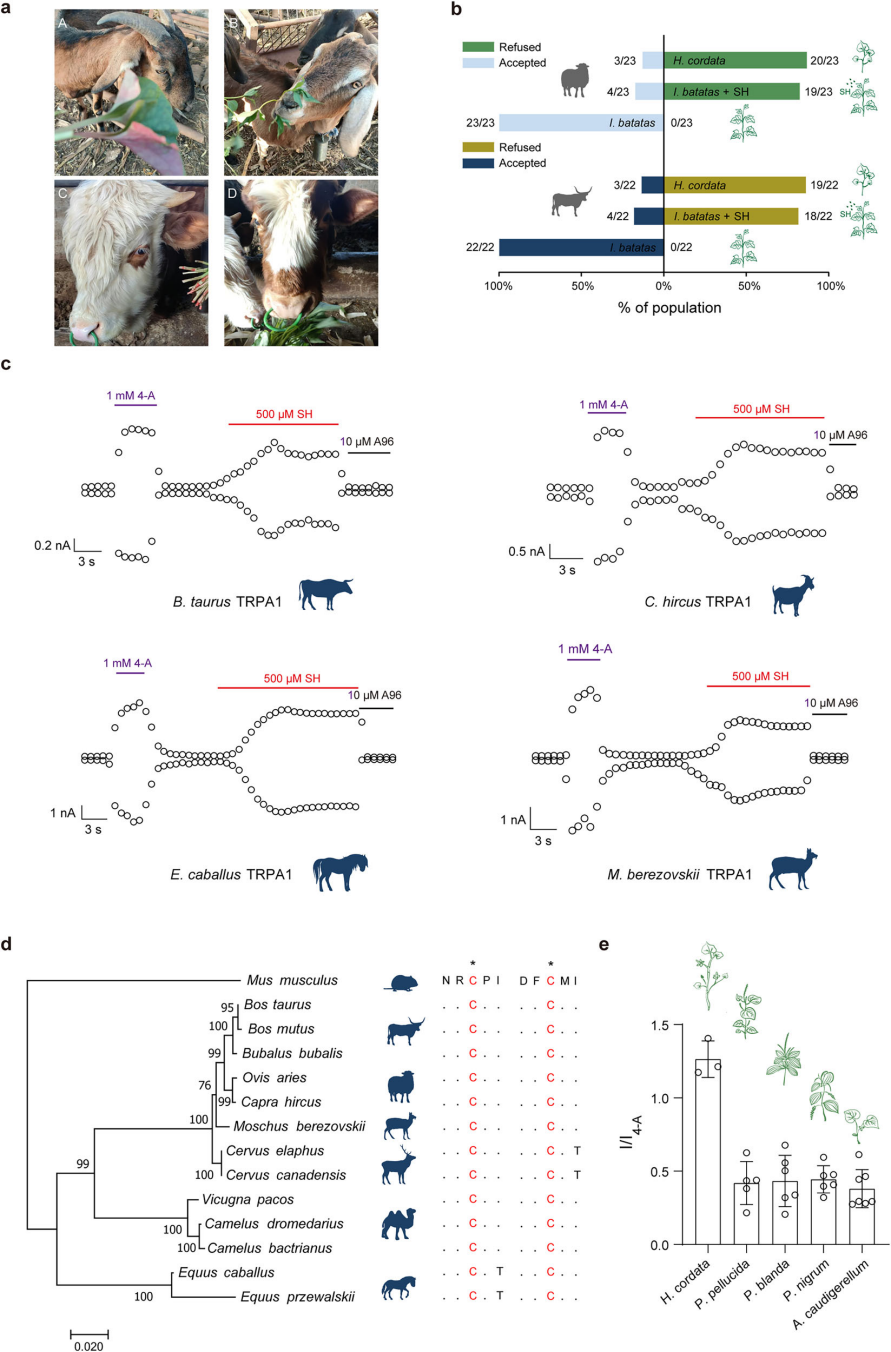
Activation of TRPA1 receptors in herbivorous mammals by leaf volatile components and species specific TRPA1 ortholog responses. **a** Representative images of goats (A) and cattle (C) refusing *H. cordata* and goats (B) and cattle (D) feeding on sweet potato leaves. **b** Comparison of feeding trials using sweet potato leaves, *H. cordata* and sweet potato leaves sprayed with 1 mM SH. Twenty-three goats and 22 cattle were tested with all leaf types twice to ensure reproducibility. Values in (**b**) are presented as rejection ratios (numerator = rejection counts; denominator = total plant presentations), with stimulus presentation sequences fully randomized per animal and trial to eliminate order effects. **c** Representative whole-cell currents from *bt*TRPA1 (*B. taurus*), *ch*TRPA1 (*C. hircus*), *ec*TRPA1 (*E. caballus*), or *mb*TRPA1 (*M. berezovskii*) were separately elicited using 500 μM SH and were inhibited by 10 μM A96. Currents were recorded at a holding potential of 0 mV, with test potentials of +80 and −80 mV. Positive control currents were elicited using 1 mM 4-A. **d** Maximum-likelihood phylogenetic tree constructed from multiple sequence alignment of TRPA1 channels in herbivores, with bootstrap values marked on nodes (1000 bootstraps). Scale bar indicates 0.02 amino acid substitutions per site. Alignment of TRPA1 amino acid sequences in herbivores, highlighting key residues associated with SH activation (orange). **e** Ratio of TRPA1 currents activated by crude extracts from representative Piperales plants compared to TRPA1 currents activated by 4-A (*n* = 3~7).

Houttuynin, the predominant volatile component in *H. cordata*, was evaluated for its role in herbivore deterrence through feeding trials involving cattle and goats (Fig. 5a). These trials demonstrated a marked aversion to *H. cordata* in both species, whereas other plant materials did not elicit comparable responses (Fig. 5b and Supplementary Video 1-2). In particular, 23 goats were presented with sweet potato (*Ipomoea batatas* (L.) Lam.) leaves across 46 feeding trials, with no significant avoidance observed for food source. However, when exposed to *H. cordata*, only 3 of the 23 goats consumed it in the initial test, while they refrained from eating it in subsequent tests. Moreover, the vast majority of the goats (20 out of 23) typically rejected the plant upon first contact. A similar aversion pattern was observed in cattle; all 22 cattle readily consumed sweet potato leaves across 44 feeding trials, while 86.36% (19 out of 22) of the cattle did not consume *H. cordata* (Fig. 5b). Consistent with the repellent effect of *H. cordata*, sweet potato leaves sprayed with SH also exhibit a significant repellent effect on goats and cattle (Fig. 5b). Specifically, in 46 tests conducted on 23 goats, 4 goats consumed the SH-sprayed sweet potato leaves during the first test, but none of them ate them again in the subsequent tests. Likewise, in 44 tests carried out on 22 cattle, 4 cattle consume the SH-sprayed sweet potato leaves in the initial test, but they also refrained from consuming them in the later tests. In conclusion, these findings indicate that houttuynin, the main component of *H. cordata*, can effectively deter the feeding behavior of herbivorous mammals.

### SH activates herbivorous TRPA1 orthologs

To further explore the molecular basis of this aversion, electrophysiological studies were conducted to assess the impact of SH, a major component of *H. cordata*, on TRPA1 channels in herbivores. 500 μM SH strongly activated TRPA1 channels in representative species, including horse (*Equus caballus*), cattle (*Bos taurus*), goat (*Capra hircus*), and forest musk deer (*Moschus berezovskii*) (Fig. 5c). This suggests that TRPA1 activation is a key molecular mechanism underlying the deterrent effects of *H. cordata* on herbivores. Further analysis explored the roles of TRPA1 residues C621 and C641 in mediating SH-induced activation. Sequence alignment of TRPA1 channels across herbivorous mammals revealed that both residues were highly conserved (Fig. 5d). This conservation implies a shared molecular strategy used by plants in the order Piperales (Fig. 5e) to defend against herbivorous mammals.

## Discussion

Plants have evolved sophisticated defense strategies to counter herbivory^5^, which play a pivotal role in maintaining ecological balance between plants and herbivores and promoting species within ecosystems. This study identifies a conserved chemical defense mechanism within the order Piperales, whereby volatile organic compounds (VOCs) deter herbivorous mammals. Our findings demonstrate that *H. cordata*, a representative species within Piperales, employs VOCs as a chemical defense, with houttuynin identified as a key component driving this repellent activity.

The volatile compounds of *H. cordata* were shown to activate TRPA1 channels in the TG of mice, inducing robust avoidance behavior. Notably, this effect was not present in TRPA1-KO mice, underscoring the specificity of the interaction and suggesting that TRPA1 activation is central to the repellent effects mediated by *H. cordata*. Functional studies across Piperales indicated that TRPA1 activation was a conserved feature of their chemical defense strategy, with key TRPA1 activation residues highly conserved among herbivorous mammals. The ability of plants to exploit conserved sensory pathways in herbivores, such as TRPA1 channels, underscores the intricate and co-evolutionary dynamics shaping plant-herbivore interactions.

Extensive research has advanced our understanding of plant defense mechanisms, focusing on specific interactions between individual plants and their herbivores, as well as the resistance of plants to herbivory through physical (e.g., thorns and spines) and chemical defenses (e.g., secondary metabolites). However, our findings reveal a distinct strategy employed by the order Piperales, which release VOCs to repel herbivorous mammals. This discovery underscores the critical role of VOCs in plant defense and expands the framework for understanding plant-herbivore interactions. Furthermore, houttuynin, a major VOC in *H. cordata*, was shown to induce aversion behaviors in herbivores by activating the TRPA1 channel. Although the role of shared evolution in plant chemical defenses has been studied previously^37^, our research provides a compelling example of shared evolution in chemical defenses. Notably, the conservation of TRPA1 activation sites across diverse herbivores suggests a universal molecular defense strategy within Piperales. These findings provide a novel molecular mechanism by which plants exploit conserved sensory pathways in herbivores to deter feeding. The role of TRPA1 activation as a molecular defense strategy highlights an innovative avenue in plant chemical defenses, where volatile signals directly influence herbivore behavior.

Early investigations into plant defense mechanisms largely centered on interactions between individual plant and herbivore species. Broadening this perspective, our research revealed a cross-species defense mechanism involving the TRPA1 channel in multiple herbivores, including cattle, goat, horse, and deer. Using electrophysiological and molecular biology techniques, VOCs were shown to interact with specific amino acid residues (C621 and C641) on the TRPA1 channel, providing novel insights into the mechanism underlying TRPA1 channel activation and advancing our understanding of plant-herbivore interactions. We further investigated TRPA1’s role in nasal mucosa, underscoring the critical involvement of trigeminal chemosensation in plant defense. Notably, our results demonstrated that plants can influence herbivore sensory pathways by leveraging olfactory trigeminal chemosensation to deter feeding, offering a novel perspective on the strategies employed by plants to enhance their survival.

While this research extensively examined *H. cordata* and its primary volatile components to elucidate chemical defense strategies in Piperales, our analysis carries important caveats. First, it relies on published empirical studies that likely underrepresent lesser-studied genera. Second, methodological variations across studies complicate cross-species comparisons, and absence of VOC reports does not equate to biological non-emission. Critically, the full range of volatile compounds produced by other species within the order remains unexplored. Given the chemical diversity of Piperales, additional and unidentified volatile compounds are likely to contribute to plant defense mechanisms. In conclusion, this study identified a conserved mechanism by which Piperales plants release volatile compounds to activate the TRPA1 channel to defend against herbivorous mammals. These findings not only deepen our theoretical understanding of plant chemical defense strategies and shared evolution but also provide practical applications for plant breeding and ecological conservation, thus advancing our understanding of animal sensory perception and plant chemical defense.

## Methods

### Materials

Sodium houttuyfonate (CAS: 83766-73-8) was purchased from Solarbio. Menthol (CAS: 2216-51-5), allyl-isothiocyanate (CAS: 57-06-7), 2-aminoethyl diphenylborinate (CAS: 524-95-8), dithiothreitol (CAS: 3483-12-3), and capsaicin (CAS: 404-86-4) were purchased from Sigma-Aldrich (USA). 4-aminodiphenylamine (CAS: 101-54-2) was purchased from Acmec (CHA). RN-1747, A-967079 and Glycine were purchased from MedChemExpress (USA).

### Animals and ethics statement

The study involving all animal studies of mice, cattle and goats conformed to the recommendations in the Guide for the Care and Use of Laboratory Animals of the Kunming Institute of Zoology, Chinese Academy of Sciences. The present study was approved by the Research Ethics Committee of Kunming Institute of Zoology, Chinese Academy of Sciences (IACUC-RE-2021-09-004). We have complied with all relevant ethical regulations for animal use. C57BL/6J mice (6–8 weeks old, Female) were purchased from the Laboratory Animal Research Center of Kunming Medical University, China. TRPA1-KO mice (6–8 weeks old, Female) were housed in the animal services facility of the Kunming Institute of Zoology under sterile conditions. The cattle (7–8 years old, Female) and goat (4–5 years old, Female) were raised on the farm. The mice were maintained in a breeding room under a 12-h light-dark cycle at 24 °C. For the isolation of TG neurons, mice were anaesthetized with 100% CO_2_ inhalation and euthanized by cervical dislocation. During the aversive taste tests, wild-type (WT) and TRPA1-KO mice were randomly designated to different experimental groups. Aqueous solutions containing sodium houttuyfonate (SH) were prepared specifically for the experiments.

### Data source and phylogenetic tree construction

We utilized the NCBI Taxonomy database (www.ncbi.nlm.nih.gov/taxonomy) to construct a phylogenetic tree of Piperales. Specifically, retrieve taxonomic classifications and hierarchical relationships for target organisms from the NCBI Taxonomy database, which integrates curated nomenclature and phylogenetic data from published sources. Export taxonomic IDs or scientific names to extract corresponding molecular sequences (e.g., via GenBank) for alignment. Alternatively, download precomputed taxonomic trees in Newick format for divergence analysis. Combine with sequence-based methods (e.g., MEGA) to validate topology or calibrate divergence times, leveraging NCBI’s standardized taxonomy to resolve nomenclature conflicts.

Subsequently, we pruned the taxa to retain only a single representative for each genus within the Piperales order. The same methodology was applied to the Oxalidales phylogeny, resulting in a streamlined representation of both plant orders. To gather relevant literature, we conducted a comprehensive search using PubMed (https://pubmed.ncbi.nlm.nih.gov) and Web of Science (https://www.webofscience.com). The search was confined to publications dating from 1990 to December 2024. The search terms were meticulously combined, incorporating the genus names of both Piperales and Oxalidales, along with the term “volatile”. This strategic combination of search terms aimed to retrieve literature that specifically addressed volatile compounds associated with the genera in question. The species mentioned in the retrieved literature were systematically recorded and presented in Supplementary Data 1.

### Extraction of VOCs

Fresh plants were collected from Kunming, Yunnan Province, China. Leaves and stems were harvested, washed within 12 h, and dried in the shade before being finely chopped. A 0.5-kg sample was weighed and added to a flask containing 1.5 L of 60% ethanol, then subjected to reflux extraction at 60 °C for 4 h. The extract was filtered to obtain the first liquid phase. The residue was then mixed with another 1.5 L of ethanol (60%) for a second reflux extraction under identical conditions. The resulting lighter orange liquid was filtered, and the two extracts were combined. The mixture was concentrated under pressure and freeze-dried to produce the crude extract. The crude extract was dissolved in ethanol and prepared for the subsequent test.

### Evaluation of plant repellent effects on herbivores

To ascertain the efficacy of the scent from *H. cordata* leaves in deterring local mammalian herbivores, we conducted feeding preference trials involving cattle (*Bos taurus* L.) and goats (*Capra hircus* L.). Throughout this study, the term “cattle” is employed to denote individuals of *B. taurus*. The experimental materials comprised the following plant leaves: fresh corn (*Zea mays* L.) leaves, sweet potato (*Ipomoea batatas* (L.) Lam.) leaves, and *H. cordata* leaves. The cattle and goats were permitted to interact freely with the array of plant leaves, with no external interference. To reduce initial alertness and potential bias, the introduction of fresh corn and sweet potato leaves was prioritized, and each subject-cattle or goat was subjected to the feeding trials on two separate occasions.

### Cell culture and transfection

Human embryonic kidney (HEK293T) cells were obtained from the Kunming Cell Bank, Kunming Institute of Zoology, Chinese Academy of Sciences. The cells were cultured in a 5% CO_2_ incubator at 37 °C in Dulbecco’s Modified Eagle Medium (DMEM) (Corning, USA) supplemented with 10% fetal bovine serum (FBS) (Corning, USA) and penicillin (100 U/mL)/streptomycin (0.1 mg/mL) (Solarbio, CHA). Transient transfection was performed using Lipofectamine 3000 (Invitrogen, USA) to express human TRPA1, TRPV1, TRPV2, TRPV3, TRPM8, ASIC2a, OTOP1 and TRPA1 from different species (including *B. taurus*, *C. hircus*, *E. caballus*, and *M. berezovskii*) plasmids, along with the pEGFPN1 plasmid. After 24 h of incubation, the transfected cells were used for patch-clamp recording.

### Electrophysiology

Electrophysiological recordings were conducted at room temperature (~22 °C) using a HEKA EPC10 amplifier and Patch Master software. Patch-clamp pipettes, with resistances of 3–5 MΩ when filled with intracellular solution, were used throughout the experiments. The bath and intracellular solutions contained (in mM): 130 NaCl, 3 HEPES, and 0.2 EDTA (pH 7.4). For whole-cell recordings, the membrane potential was clamped at 0 mV, and currents were elicited using a 250-ms step to +80 mV, followed by a 250-ms step to −80 mV at 1-s intervals. For inside-out and outside-out recordings, the membrane potential was also clamped at 0 mV, and currents were induced using 500-ms steps to either +80 mV or −80 mV. Solutions were applied to the membrane patch using a gravity-driven perfusion system (RSC-200) (Bio-Logic, FRA). The same patch-clamp protocols were applied for TRPV1, TRPV2, TRPV3, and TRPM8 channels as those used for TRPA1 channels. For ASIC2a channels, the bath solution contained (in mM): 150 NaCl, 5 KCl, 1 MgCl_2_, 2 CaCl_2_, 10 glucose, and 10 HEPES (pH 7.4), while the intracellular solution contained (in mM): 120 KCl, 30 NaCl, 1 MgCl_2_, 0.5 CaCl_2_, 5 EGTA, 2 Mg^2+^-ATP, and 10 HEPES (pH 7.25). For OTOP1 channels, the bath and intracellular solutions contained (in mM): 160 NMDG-Cl, 2 CaCl_2_, and 10 HEPES (pH 7.4). The above reagents were obtained from Sigma-Aldrich (USA).

### Clones and mutagenesis

Human TRPA1 (NM_007332), TRPV1 (NM_080706), TRPV2 (NM_016113), TRPV3 (NM_145068), TRPM8 (NM_001397617), ASIC2a (NM_001094), OTOP1 (NM_177998), *Bos taurus* TRPA1(MF063039), *Capra hircus* TRPA1 (XM_018058422), *Equus caballus* TRPA1 (XM_070631514), and *Moschus berezovskii* TRPA1 (XM_055431955) sequences were collected from NCBI. All sequences were codon-optimized for human expression, synthesized, and subcloned into the pcDNA3.1 vector by Sangon Biotech (CHA) using NheI/XhoI restriction sites. Site-directed mutagenesis was performed using an oligonucleotide-based approach with the Mut-Express-II-Fast-Mutagenesis Kit-V2 (Vazyme, CHA). All mutations were confirmed by DNA sequencing.

### Genotype identification

For genotyping, DNA was extracted from tail snips using the Mouse Direct PCR Kit (Selleck, USA). PCR amplification was performed using the following primers: Forward primer: 5’-GCCTTTCTTGAACTCTCACC-3’; Reverse primer: 5’-ATCACCTACCAGTAAGTTCATCAGAAGA-3’. These primers were designed to detect either the WT TRPA1 gene (~300 bp product) or the TRPA1 pore loop deletion gene (~200 bp product).

### Isolation and culture of TG neurons

Newborn WT and TRPA1-KO mice were euthanized, and the TG tissue was dissociated and washed with Hank’s buffer (Gibco, USA). The tissue was then minced into small pieces and digested in a solution containing collagenase I and trypsin for 30 min at 37 °C. A trypsin inhibitor was added, and the suspension was centrifuged at 3000 rpm for 10 min. The resulting pellet was washed twice with pre-cooled phosphate-buffered saline (PBS) and once with 1 mL of cell culture medium. Cells were resuspended in Neurobasal A (Invitrogen, USA) containing 2% B27 (Invitrogen, USA) and seeded onto round cover slips in 35-mm culture dishes. The dishes were incubated at 37 °C for further experiments.

### Calcium imaging

TG neurons or HEK293T cells were stained with Fluo-4 AM (Invitrogen, USA) in 2 mM Ca^2+^ Ringer’s solution. The Ringer’s solution contained (in mM):140 NaCl, 5 KCl, 2 MgCl_2_, 10 glucose, 2 CaCl_2_, and 10 HEPES (pH 7.4). Fluorescence was visualized using an Olympus IX81 microscope equipped with a Hamamatsu R2 charge-coupled device camera controlled by MetaFluor Software (Molecular Devices, USA). Fluo-4 was excited using an LED light source with a 500/20 nm excitative filter, and fluorescence emission was detected through a 535/30 nm emission filter. Fluorescence graphs were constructed using MetaMorph software (Molecular Devices, USA) and analyzed using Igor Pro (WaveMetrics, USA).

### Two-bottle taste preference test

The experiment consisted of four groups, each with eight replicates. WT C57BL/6J mice and TRPA1-KO C57BL/6J mice were reared in individual cages for 6 days. During the experiment, water was supplied using a custom-built container. The container comprised a stainless-steel pipette inserted into a 10-mL serum pipette, with the opposite end sealed with a rubber plug and sealing film. The device was positioned symmetrically within the cage to eliminate positional bias. To further control side preferences, the positions of the two containers were switched every 24 h. During the test, mice were provided with one tube containing deionized water and another containing an aqueous solution of SH for 3 h daily. Drinking volume was measured and the experiment was carried out for six consecutive days. The results were quantified by calculating the total volume of SH solution consumed divided by the total volume of water consumed during the experiment.

### Establishment of olfactory deprivation model

The olfactory deprivation model was developed following methods previously described^38^. Initially, the food finding test (FFT) olfactory paradigm, as described by Deacon et al.^39^, was employed to identify mice exhibiting normal olfactory capabilities. Prior to testing, the mice underwent a 24-h food deprivation period. The assessment was conducted under dim white light (20 lx) during the light phase of the light/dark cycle, utilizing a novel cage (dimensions: 45 cm × 24 cm × 20 cm) containing 3 cm of fresh bedding. A cookie weighing 45 mg was placed at one end of the cage and concealed beneath 1 cm of bedding. The mouse was positioned in a corner of the cage, oriented towards the walls, and its behavior was meticulously observed. The latency period was defined as the duration from the introduction of the mouse into the cage until the discovery of the food pellet, with mice failing to locate the food within 10 minutes classified as having impaired olfactory function. Each mouse was tested individually in a sanitized cage. Mice demonstrating normal olfactory function were anesthetized with isoflurane and administered a nasal infusion of 5 µL of chloroform in each nostril, subsequently being randomly assigned to one of three groups. The olfactory deprivation behavior was assessed 24 hours post-chloroform treatment using the FFT, followed by two-bottle taste preference test involving exposure to water, 1000 μM SH, and 200 μM AITC, respectively.

### Nasal mucosa isolation

Following euthanasia, the murine nasal mucosa is isolated by first bluntly dissecting and reflecting the head skin to expose the skull; the zygomaticomaxillary junctions are then severed bilaterally with scissors, and the nasal bones are elevated via the nares using forceps. Subsequently, soft tissue overlying the hard palate is dissected to expose the nasal cavity floor, followed by fracturing the bilateral maxillary alveolar bone with forceps and excising the incisors and bony nasal tip with scissors. The bony nasal septum’s ventral attachments to the maxillae are transected with a scalpel or scissors; the anterior maxillae are retracted laterally to open the nasal cavity, and the bony septum is grasped at its base, detached from cranial attachments (notably the ethmoid), and carefully removed while ensuring mucosal integrity, ultimately exposing the mucosa lining the maxillary bones (lateral walls) and the septal mucosa (medial walls) for harvest.

### Quantitative real-time PCR

Total RNA was extracted using trizol reagent (Invitrogen, USA). RNA was reverse transcribed into cDNA using the 5× All-In-One MasterMix Kit (Abm, Canada). Real-time Quantitative PCR was performed with the BlasTaq 2× qPCR MasterMix (Abm, Canada) and the Step One Plus Real-Time PCR system (Thermo, USA). Expression levels were normalized using the 2^ΔΔ^CT method with GAPDH as the reference gene. The primers sequences were TRPA1 Forward: AATCTCTGTCCTCTGCATCACG; TRPA1 Reverse: ACAATGCAGTGGGGTATTTCC; GAPDH Forward: GAAGGTCGGTGTGAACGGAT； GAPDH Reverse: AATCTCCACTTTGCCACTGC.

### Statistics and reproducibility

Animal and specimen sample sizes were selected to ensure statistical robustness and were consistent with those in similar studies. Animals were randomly assigned to experimental groups. Data normality was assessed using the Shapiro–Wilk method, while variance equality between groups was measured using F-tests or Levene’s tests. Statistical significance was determined using one-way analysis of variance (ANOVA), followed by Bonferroni’s or Sidak’s tests for multi-group comparisons, performed with GraphPad Prism v7 (GraphPad Software Ltd., USA). Results are expressed as the mean ± standard error of the mean (SEM), with statistical significance defined as *P* < 0.05. Non-significant differences are denoted as “ns”.

## Supplementary information

Supplemental Information

Description of Additional Supplementary Files

Supplementary Data 1

Supplementary Data 2

Supplementary Video 1

Supplementary Video 2

## Data Availability

The data supporting this study are available from the corresponding authors on reasonable request. All data are available within the article and within the Supplementary Information published online. The numerical source data for graphs are available in Supplementary Data 2. Uncropped and unedited blot/gel images (Supplementary Fig. 7) are provided in the Supplementary Information.

## References

[1] Weis, A. E. & Franks, S. J. Herbivory tolerance and coevolution: an alternative to the arms race?. *N. Phytol.* **170**, 423–425 (2006). 10.1111/j.1469-8137.2006.01745.x 16626464

[2] Endara, M. J. et al. Coevolutionary arms race versus host defense chase in a tropical herbivore-plant system. *Proc. Natl Acad. Sci. USA* **114**, E7499–e7505 (2017). 28827317 10.1073/pnas.1707727114 PMC5594685

[3] Cloern, J. E., Canuel, E. A. & Harris, D. Stable carbon and nitrogen isotope composition of aquatic and terrestrial plants of the San Francisco Bay estuarine system. *Limnol. Oceanogr.* **47**, 713–729 (2002).

[4] Turley, N. E., Godfrey, R. M. & Johnson, M. T. J. Evolution of mixed strategies of plant defense against herbivores. *N. Phytol.* **197**, 359–361 (2013). 10.1111/nph.12103 23253329

[5] Mithöfer, A. & Boland, W. Plant defense against herbivores: chemical aspects. *Annu Rev. Plant Biol.* **63**, 431–450 (2012). 22404468 10.1146/annurev-arplant-042110-103854

[6] Hanley, M. E., Lamont, B. B., Fairbanks, M. M. & Rafferty, C. M. Plant structural traits and their role in anti-herbivore defence. *Perspect. Plant Ecol. Evol. Syst.* **8**, 157–178 (2007).

[7] Pare, P. W. & Tumlinson, J. H. Plant volatiles as a defense against insect herbivores. *Plant Physiol.* **121**, 325–332 (1999). 10517823 PMC1539229

[8] Heil, M. Herbivore- induced plant volatiles: targets, perception and unanswered questions. *N. Phytol.* **204**, 297–306 (2014).

[9] Turlings, T. C. J. & Wäckers, F. Recruitment of predators and parasitoids by herbivore-injured plants. In *Advances in Insect Chemical Ecology*, 21–75 (Cambridge University Press, 2004).

[10] Mertens, D. et al. Plant defence to sequential attack is adapted to prevalent herbivores. *Nat. Plants* **7**, 1347–1353 (2021). 34650263 10.1038/s41477-021-00999-7

[11] War, A. R. et al. Mechanisms of plant defense against insect herbivores. *Plant Signal Behav.* **7**, 1306–1320 (2012). 22895106 10.4161/psb.21663 PMC3493419

[12] Qiao, X., Zhang, S. & Paterson, A. H. Pervasive genome duplications across the plant tree of life and their links to major evolutionary innovations and transitions. *Comput. Struct. Biotechnol. J.* **20**, 3248–3256 (2022). 35782740 10.1016/j.csbj.2022.06.026 PMC9237934

[13] Jaramillo-Colorado, B. E., Pino-Benitez, N. & Gonzalez-Coloma, A. Volatile composition and biocidal (antifeedant and phytotoxic) activity of the essential oils of four Piperaceae species from Choco-Colombia. *Ind. Crops Products* **138**, 10.1016/j.indcrop.2019.06.026 (2019).

[14] Wu, Z. et al. Houttuynia cordata Thunb: An Ethnopharmacological Review. *Front. Pharmacol.* **12**, 10.3389/fphar.2021.714694 (2021). 10.3389/fphar.2021.714694 PMC8440972 34539401

[15] Huang, P. et al. A genome assembly of decaploid Houttuynia cordata provides insights into the evolution of Houttuynia and the biosynthesis of alkaloids. *Horticulture Res.* **11**, 10.1093/hr/uhae203 (2024). 10.1093/hr/uhae203 PMC11415239 39308792

[16] Yang, Z. et al. A near-complete assembly of the Houttuynia cordata genome provides insights into the regulatory mechanism of flavonoid biosynthesis in Yuxingcao. *Plant Commun.* **5**, 101075 (2024). 39228129 10.1016/j.xplc.2024.101075 PMC11573901

[17] Liu, X. et al. Sodium houttuyfonate: A review of its antimicrobial, anti-inflammatory and cardiovascular protective effects. *Eur. J. Pharm.* **902**, 174110 (2021). 10.1016/j.ejphar.2021.174110 33901457

[18] Doty, R. L. & Cometto-Muňiz, J. E. *Handbook of Olfaction and Gustation* 1673–1704 (CRC Press, 2003).

[19] Jordt, S. E. et al. Mustard oils and cannabinoids excite sensory nerve fibres through the TRP channel ANKTM1. *Nature* **427**, 260–265 (2004). 14712238 10.1038/nature02282

[20] Caterina, M. J. et al. The capsaicin receptor: a heat-activated ion channel in the pain pathway. *Nature* **389**, 816–824 (1997). 9349813 10.1038/39807

[21] Story, G. M. et al. ANKTM1, a TRP-like channel expressed in nociceptive neurons, is activated by cold temperatures. *Cell* **112**, 819–829 (2003). 12654248 10.1016/s0092-8674(03)00158-2

[22] Kobayashi, K. et al. Distinct expression of TRPM8, TRPA1, and TRPV1 mRNAs in rat primary afferent neurons with adelta/c-fibers and colocalization with trk receptors. *J. Comp. Neurol.* **493**, 596–606 (2005). 16304633 10.1002/cne.20794

[23] Kim, Y. S., Kim, S. K., Lee, J. S., Ko, S. J. & Bae, Y. C. Expression of vesicular glutamate transporters in transient receptor potential ankyrin 1 (TRPA1)-positive neurons in the rat trigeminal ganglion. *Brain Res.* **1690**, 31–39 (2018). 29649466 10.1016/j.brainres.2018.04.010

[24] Katsura, H., Tsuzuki, K., Noguchi, K. & Sakagami, M. Differential expression of capsaicin-, menthol-, and mustard oil-sensitive receptors in naive rat geniculate ganglion neurons. *Chem. Senses* **31**, 681–688 (2006). 16831854 10.1093/chemse/bjl009

[25] Ye, Y. Z. et al. Molecular sensors for temperature detection during behavioral thermoregulation in turtle embryos. *Curr. Biol.* **31**, 2995–3003.e2994 (2021). 34015251 10.1016/j.cub.2021.04.054

[26] Viana, F. Chemosensory properties of the trigeminal system. *ACS Chem. Neurosci.* **2**, 38–50 (2011). 22778855 10.1021/cn100102c PMC3369707

[27] Cheng, Y. R., Jiang, B. Y. & Chen, C. C. Acid-sensing ion channels: dual function proteins for chemo-sensing and mechano-sensing. *J. Biomed. Sci.* **25**, 46 (2018). 29793480 10.1186/s12929-018-0448-y PMC5966886

[28] Zhang, J. et al. Sour sensing from the tongue to the brain. *Cell* **179**, 392–402.e315 (2019). 31543264 10.1016/j.cell.2019.08.031

[29] Pozsgai, G. et al. Evidence for the pathophysiological relevance of TRPA1 receptors in the cardiovascular system in vivo. *Cardiovasc. Res.* **87**, 760–768 (2010). 20442136 10.1093/cvr/cvq118

[30] Nagatomo, K. & Kubo, Y. Caffeine activates mouse TRPA1 channels but suppresses human TRPA1 channels. *Proc. Natl Acad. Sci. USA* **105**, 17373–17378 (2008). 18988737 10.1073/pnas.0809769105 PMC2582301

[31] Richards, P. M., Johnson, E. C. & Silver, W. L. Four irritating odorants target the trigeminal chemoreceptor TRPA1. *Chemosens. Percept.* **3**, 190–199 (2010).

[32] Dinkova-Kostova, A. T., Massiah, M. A., Bozak, R. E., Hicks, R. J. & Talalay, P. Potency of Michael reaction acceptors as inducers of enzymes that protect against carcinogenesis depends on their reactivity with sulfhydryl groups. *Proc. Natl Acad. Sci. USA* **98**, 3404–3409 (2001). 11248091 10.1073/pnas.051632198 PMC30666

[33] Eggler, A. L., Liu, G., Pezzuto, J. M., van Breemen, R. B. & Mesecar, A. D. Modifying specific cysteines of the electrophile-sensing human Keap1 protein is insufficient to disrupt binding to the Nrf2 domain Neh2. *Proc. Natl Acad. Sci. USA* **102**, 10070–10075 (2005). 16006525 10.1073/pnas.0502402102 PMC1177374

[34] Macpherson, L. J. et al. Noxious compounds activate TRPA1 ion channels through covalent modification of cysteines. *Nature* **445**, 541–545 (2007). 17237762 10.1038/nature05544

[35] Getz, E. B., Xiao, M., Chakrabarty, T., Cooke, R. & Selvin, P. R. A comparison between the sulfhydryl reductants tris(2-carboxyethyl)phosphine and dithiothreitol for use in protein biochemistry. *Anal. Biochem.* **273**, 73–80 (1999). 10452801 10.1006/abio.1999.4203

[36] Paulsen, C. E., Armache, J. P., Gao, Y., Cheng, Y. & Julius, D. Structure of the TRPA1 ion channel suggests regulatory mechanisms. *Nature* **520**, 511–517 (2015). 25855297 10.1038/nature14367 PMC4409540

[37] Negin, B. & Jander, G. Convergent and divergent evolution of plant chemical defenses. *Curr. Opin. Plant Biol.* **73**, 102368 (2023). 37087925 10.1016/j.pbi.2023.102368

[38] Zheng, X. et al. Bilateral olfactory mucosa damage induces the disappearance of olfactory glomerulus and reduces the expression of extrasynaptic α5GABAARs in the hippocampus in early postnatal sprague dawley rats. *Neurotox. Res.* **34**, 353–362 (2018). 29667127 10.1007/s12640-018-9893-3

[39] Deacon, R., Koros, E., Bornemann, K. & Rawlins, J. Aged Tg2576 mice are impaired on social memory and open field habituation tests. *Behav. Brain Res.* **197**, 466–468 (2009). 18977397 10.1016/j.bbr.2008.09.042

