# Supplementary material for: Molecular defense strategy of volatile organic compound-emitting plants (order Piperales) against herbivorous mammals
Source: Commun Biol. 2025 Dec 1;9:19. doi: 10.1038/s42003-025-09273-4 (PMC12770358; doi:10.1038/s42003-025-09273-4)
Supplement: Supplementary file 1 — Supplemental Information [file 42003_2025_9273_MOESM1_ESM.pdf]

Supplemental Figures

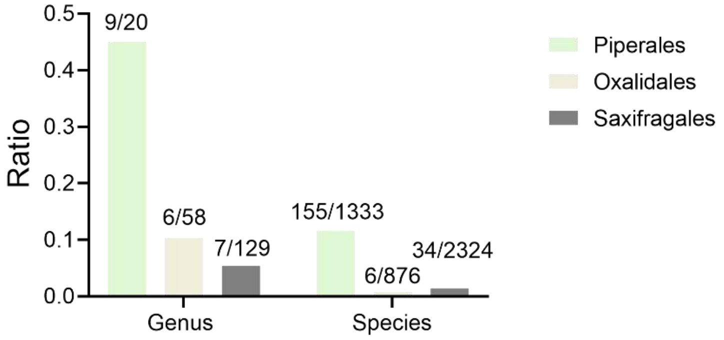

Supplemental Figure 1 Proportion of VOC-emitting plants at species and genus levels. Values are presented as ratios (numerator = volatile plant count; denominator = total plant assessed).

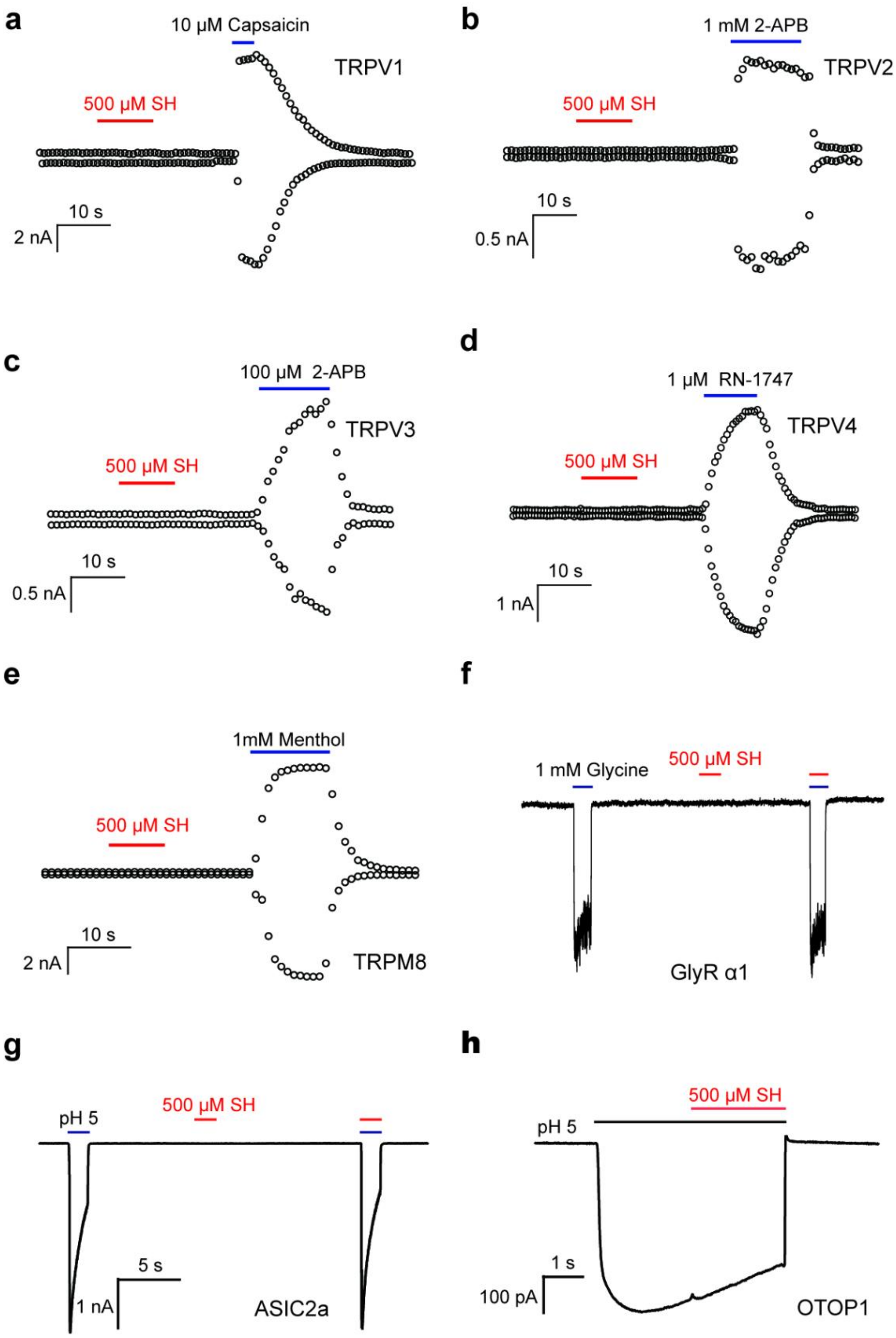

Supplemental Figure 2 Selectivity of SH on chemosensation-related ion channels. (a-h) Selectivity of 500 μM SH evaluated on various chemosensation-related ion channels, including TRPV1 (a), TRPV2 (b), TRPV3 (c), TRPV4 (d), TRPM8 (e), GlyR α1 receptor (f), ASIC2a (g), and OTOP1 (h). Recordings were conducted with 6-8 cells for each channel type.

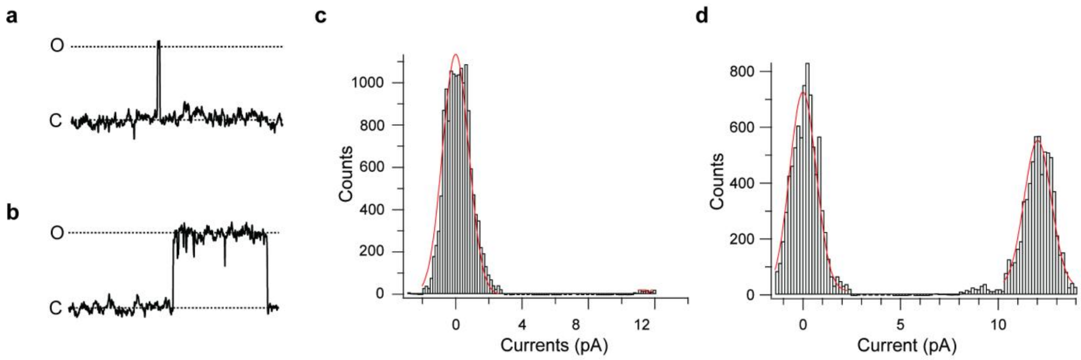

Supplemental Figure 3 Potentiation of TRPA1 channel by SH at the single-channel level. (a, b) Representative single-channel current traces recorded at +80 mV in the absence (a) and presence (b) of 500 μM SH. (c, d) All-point histograms of single-channel events recorded in bath solution (c) and with 500 μM SH (d). Superimposed curves represent best-fit analysis based on a double-Gaussian distribution model.

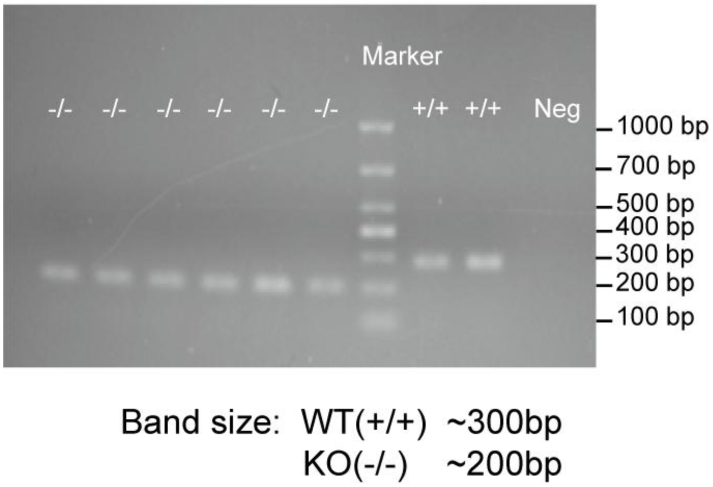

Supplemental Figure 4 Genotyping of TRPA1-KO mice. Neg indicates negative control. -/- indicates TRPA1-KO mice, +/+ indicates WT mice.

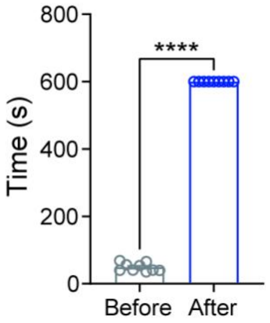

Supplemental Figure 5 Wild-type mice with intact olfaction located the buried food rapidly (approximately 48.9 s), whereas surgically treated mice failed to find the food within the 10-minute test period and were classified as olfactory-impaired (n = 9).

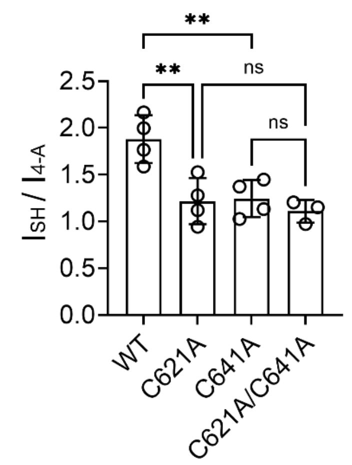

Supplemental Figure 6 Normalized currents calculated as the ratio of SH-induced currents to 4-A-induced currents in response to 500 μM SH in HEK293 cells expressing WT hTRPA1 or hTRPA1 mutants ($n = 3$~4). Statistical analysis was conducted using one-way ANOVA ($F = 9.724$), followed by Sidak's multiple comparisons test: WT vs. C 621A, $t = 4.281$, $p = 0.0052$; WT vs. C641A, $t = 4.102$, $p = 0.0070$. C621A vs. C621A/ C641A, $t = 0.6439$, $p = 0.9524$; C641A vs. C621A/ C641A, $t = 0.8100$, $p = 0.8982$; **$p < 0.01$, n.s., no significant difference. Data are presented as mean ± SEM.

Uncropped and unedited blot/gel images

1. Images related to Supplemental Figure 4

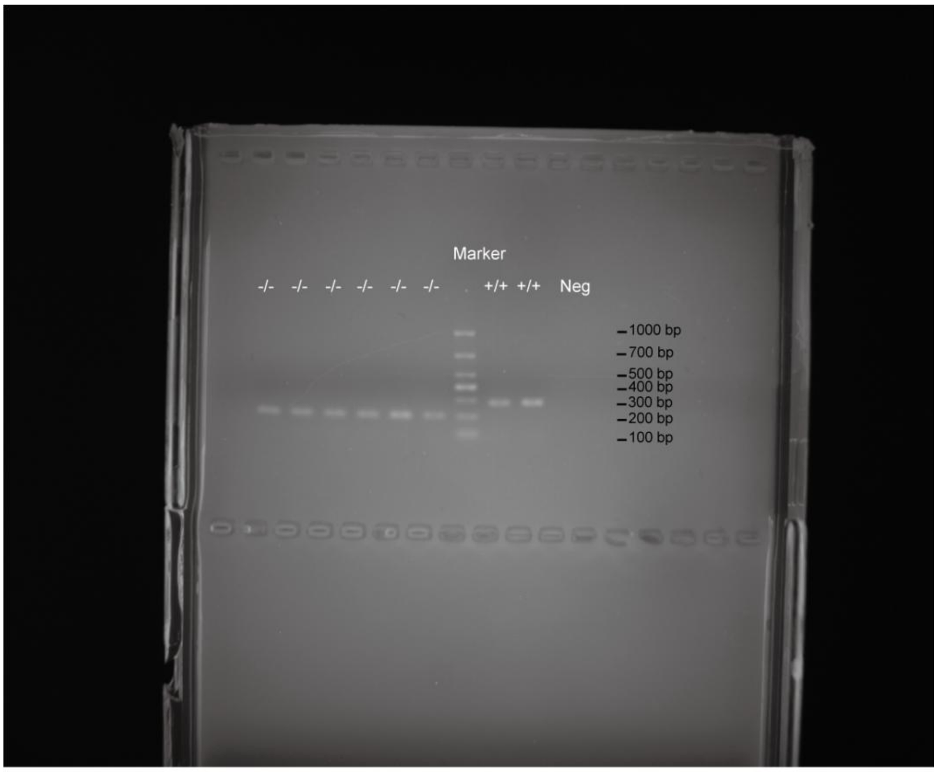

Band size: WT(+/+) ~300bp
KO(-/-) ~200bp

Supplementary Figures 7 Uncropped images corresponding to Supplemental Figure 4
