# Supplementary material for: Molecular defense strategy of volatile organic compound-emitting plants (order Piperales) against herbivorous mammals
Source: Commun Biol. 2025 Dec 1;9:19. doi: 10.1038/s42003-025-09273-4 (PMC12770358; doi:10.1038/s42003-025-09273-4)
Supplement: Supplementary file 2 — Description of Additional Supplementary Files [file 42003_2025_9273_MOESM2_ESM.pdf]

Description of Additional Supplementary Files

File name: Supplementary Data 1:
Description: Literature retrieval statistics in Fig. 1a

File name: Supplementary Movie 1:
Description: Cow takes Ipomoea batatas leaves

File name: Supplementary Movie 2:
Description: Cow refused to take Houttuynia cordata
